# We Should Have Talked About Hospice: Transforming Communication With Bereaved LGB Aging Women

**DOI:** 10.1093/geroni/igab046.2037

**Published:** 2021-12-17

**Authors:** Korijna Valenti, Leah Janssen

**Affiliations:** 1 University of Colorado--Denver, Denver, Colorado, United States; 2 Scripps Gerontology Center, Oxford, Ohio, United States

## Abstract

Because of historical discrimination, discomfort disclosing information, and differing definitions of family, lesbian, gay, bisexual, and transgender (LGBT) older adults with serious illness need both improved palliative and end-of-life (EOL) care communication with clinicians and recognized inclusion of spouses/partners. Communicating about palliative and EOL care may improve the care goals and emotional trajectory for patients and significant others. Using a descriptive qualitative approach, this study’s aim was to analyze the communication experiences during a spouse’s/partner’s EOL care for bereaved LGB women (n=16) 60 and older. Drawing on queer gerontology, issues relating to access to resources and information and the systemic silencing of older LGB women illuminate areas where policy and practice may be improved. Semi-structured, one-on-one interviews were used to provide deep and meaningful information about palliative and EOL care communication between participants, their spouse or partner, and clinicians. While results reflect certain outcomes found in prior studies with non-LGBT adults, thematic analysis revealed three main findings with evidence specific to this population: 1) avoiding deep discussions about EOL; 2) lack of understanding about palliative or EOL care; and 3) limited communication with clinicians. Findings illuminate the need for better understanding among clinicians regarding palliative and EOL communication with LGBT dyads as well as communication strategies based on recognition and acceptance. Further dyadic communication research may improve care goals for LGBT older adults. Understanding couples’ interactions and examining different communication behaviors may lead to improved palliative and EOL care goals for older LGBT adults with serious illness and their spouses/partners.

